# A young boy with ventricular arrhythmias and thyroid dysgenesis: two genes are not enough?

**DOI:** 10.20945/2359-3997000000546

**Published:** 2022-12-01

**Authors:** Roberto Franceschi, Evelina Maines, Maria Bellizzi, Francesca Rivieri, Andrea Bacca, Alessandra Filippi, Enza Maria Valente, Massimo Plumari, Massimo Soffiati, Monica Vincenzi, Francesca Teofoli, Marta Camilot

**Affiliations:** 1 S. Chiara Hospital of Trento Pediatric Department Trento Italy Pediatric Department, S. Chiara Hospital of Trento, Trento, Italy; 2 S. Chiara Hospital of Trento Genetic Unit Trento Italy Genetic Unit, S. Chiara Hospital of Trento, Trento, Italy; 3 S. Chiara Hospital of Trento Cardiology Unit Trento Italy Cardiology Unit, S. Chiara Hospital of Trento, Trento, Italy; 4 Azienda Provinciale per i Servizi Sanitari del Trentino Pediatric Neuropsychology Unit Trento Italy Pediatric Neuropsychology Unit, Azienda Provinciale per i Servizi Sanitari del Trentino, Trento, Italy; 5 IRCCS Mondino Foundation Neurogenetics Research Center Pavia Italy Neurogenetics Research Center, IRCCS Mondino Foundation, Pavia, Italy; 6 University of Pavia Department of Molecular Medicine Pavia Italy Department of Molecular Medicine, University of Pavia, Pavia, Italy; 7 University of Verona Department of Surgical, Odontostomatological, Mother and Child Sciences Verona Italy Department of Surgical, Odontostomatological, Mother and Child Sciences, University of Verona, Verona, Italy

## Abstract

Congenital hypothyroidism (CH) may be caused by biallelic variants in the TSHR gene. CH due to thyroid dysgenesis has also been linked to pathogenic variants of the nucleotide kinase 2, homeobox 5 (NKX2-5) gene, which can also cause sudden cardiac death from ventricular arrhythmia. In particular, the NKX2-5 p.Arg25Cys missense variant has been repeatedly reported in patients with congenital heart defects and, more rarely, with hypogonadism. We report the case of a 7 year old boy with ventricular arrhythmias, thyroid dysgenesis and intellectual disability, born from consanguineous Tunisian parents. Exome sequencing and segregation analysis revealed two potentially relevant variants: the NKX2-5 p.Arg25Cys variant (maternally inherited), as well as a single heterozygous TSHR p.Gln90Pro variant (paternally inherited). Of note, a male sibling of the proband, presenting with intellectual disability only, carried the same two variants. No other TSHR variants, or other potentially relevant variants were identified. In this proband, despite the identification of variants in two genes potentially correlated to the phenotype, a definite genetic diagnosis could not be reached. This case report highlights the complexity of exome data interpretation, especially when dealing with families presenting complex phenotypes and variable expression of clinical traits.

## INTRODUCTION

The human nucleotide kinase 2, homeobox 5 (*NKX2-5*) gene, located on chromosome 5q35.1 and encoding the homeobox-containing transcription factor NKX2.5, is known to be essential for normal heart morphogenesis, myogenesis and proper cardiac function (1). Several inactivating mutations in the *NKX2-5* gene, primarily located within the homeodomain, have been described in patients with a spectrum of congenital heart diseases (CHD), the most frequent ones being atrial or ventricular septal defects and tetralogy of Fallot (2). In addition, *NKX2-5* pathogenic variants have been reported in adult patients with heart conduction defects such as atrial fibrillation (3) and, more recently, with sudden cardiac death from ventricular arrhythmia (4–6).

Besides its role in cardiac development, the presence of a fully functional NKX2-5 is essential for early thyroid morphogenesis, both for regulation of thyroidal cell differentiation and for proper cell migration to the final position in the neck (7). Accordingly, heterozygous *NKX2-5* variants have also been linked to congenital hypothyroidism (CH) and thyroid dysgenesis (7–10).

It is well known that CH is associated with a significantly higher frequency of CHD (11), as well as malformations of the urinary, gastrointestinal, and skeletal systems (12). While this can be attributed, in some cases, to the metabolic derangement due to the shortage of thyroid hormones, the impairment of genes involved in heart and thyroid organogenesis, such as *NKX2-5*, may also play a crucial role (7). Besides *NKX2-5*, several other genes have been involved in thyroid dysgenesis, such as the β-subunit of TSH (*TSHB*), the TSH receptor (*TSHR)*, and another well-known transcription factor encoded by the paired-box gene 8 (*PAX8*) (13).

We report a patient with ventricular arrhythmias and thyroid dysgenesis, carrying two heterozygous variants in the *NKX2-5* and *TSHR* genes. While variants in both genes are potentially related to these phenotypes, the interpretation of genetic results can be extremely complex, as illustrated by the patient described here.

## CASE REPORT

### Clinical features

A young boy, second-born to parents from Tunisia who are first grade cousins, was referred to our pediatric department. Born at 39 weeks of gestation after an uneventful pregnancy, his birth weight was 2250 g (-2.9 SDS), length 45 cm (-2.81 SDS), and head circumference 32.5 cm (-1.91 SDS).

CH was diagnosed at neonatal screening and confirmed at 6 days of life, with serum TSH 740 mU/L (reference range < 8.8 mU/L), FT4 0.9 pmol/L (13.9-26.1 pmol/L), and FT3 1.2 pmol/L (4.5-10.5 pmol/L). L-thyroxine treatment was promptly initiated with L-thyroxine 37.5 μg daily (15 μg/kg daily). At 6 months of life, a neck ultrasound (US) revealed thyroid hemiagenesis. During follow-up, L-thyroxine dose was adjusted for age and weight and thyroid function tests were always in the reference range. At 3 years of age, he displayed intellectual disability (ID) with a total IQ of 55 (WIPPSI III scale, normal values 85-115, deficit under 70), deep language impairment in production and understanding, and severe attention deficit.

**Figure 1:**
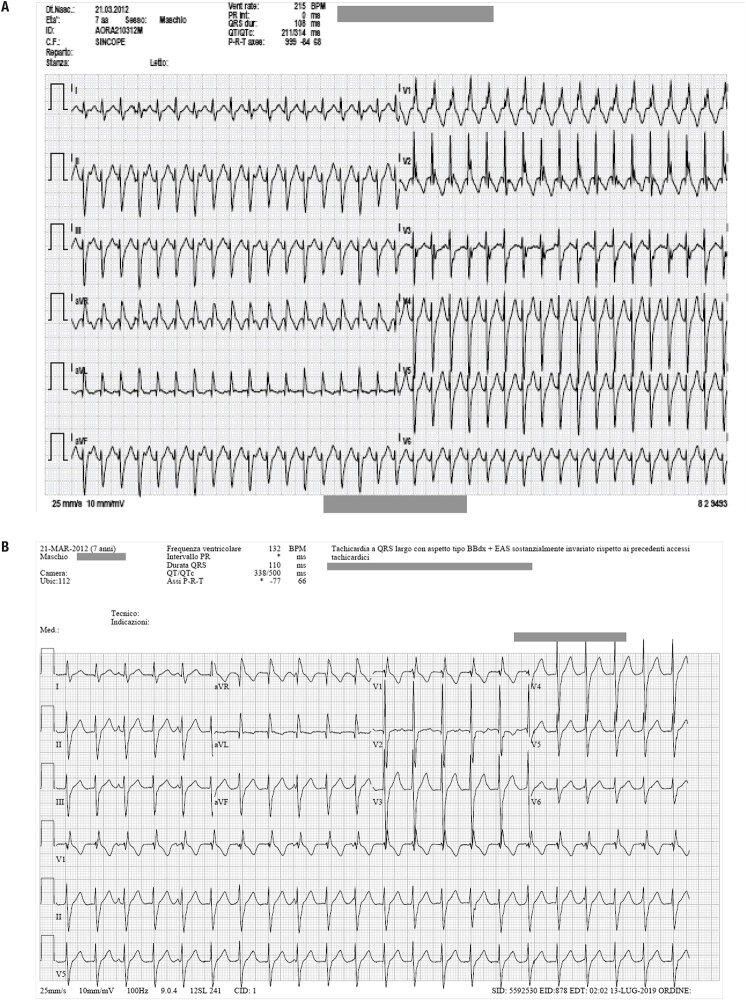
On the ECG at 7 years and 3 month of age (Figure 1A), there was a large QRS tachycardia, right bundle branch block and left anterior hemiblock. One month later (Figure 1B), he presented again ventricular fascicular tachycardia.

In June 2019, at age 7 years and 3 months, he presented to the emergency room for malaise, nausea and vomiting, with episodes of loss of consciousness. ECG showed a large QRS tachycardia, right bundle branch block and left anterior hemiblock (Figure 1A). Vagal maneuvers and repeated adenosine i.v. (0.1 mg/kg and subsequently 0.2 mg/kg) were ineffective, therefore he underwent electrical cardioversion with success. Heart US was normal. Prophylaxis with low-dose verapamil 5 mg/kg daily was started during hospitalization and Holter ECG turned out negative. One month later, he presented another episode of ventricular fascicular tachycardia (Figure 1B); verapamil dosage was increased to 11 mg/kg daily and flecainide 60 mg/m^2^ daily was started. Holter ECG showed very frequent ventricular ectopic beats (10% of the total), bimorphs isolated and in pairs, with a prevalent morphology; frequent ventricular beats to blanks, the longest of 26 beats and the fastest at a heart rate of 136 bpm.

On last examination, the patient was 9 years old; weight was -1.7 SDS and height -0.7 SDS. WISC IV psychometric test confirmed a moderate ID. He is on L-thyroxine 75 μg, verapamil 10 mg/kg and flecainide 60 mg/m^2^ daily.

**Figure 2:**
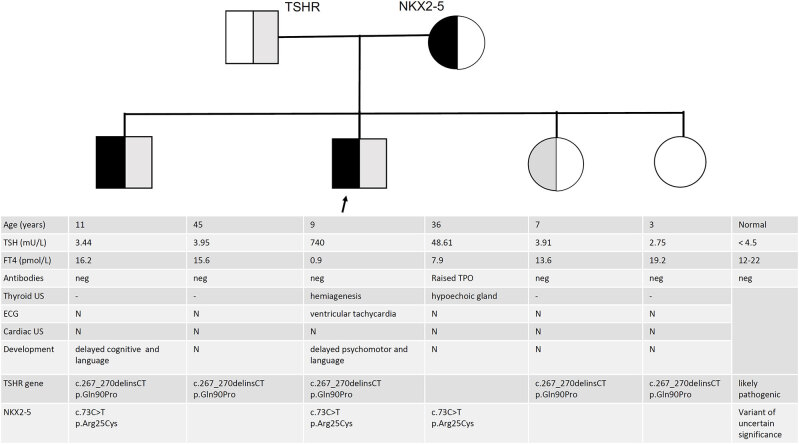
Pedigree of the family harboring the c.73C>T mut. in NKX2.5 gene. The index patient is indicated by an arrow. Squares: male; circles: female; the transmission of the NKX2.5 variant is shown by a filled black area within each symbol, whereas the segregation of TSHR variant by a corresponding grey area. Results of thyroid and heart investigations are aligned with each symbol.

The mother suffers from acquired hypothyroidism (Hashimoto thyroiditis) diagnosed after the first pregnancy in September 2010. She is on levothyroxine 75 μg/day. Her ECG and cardiac ultrasound are normal. In the father, cognitive levels, ECG and thyroid function are normal. The propositus has one brother and two sisters: the boy is the first-born (January 2010) and displays ID (total IQ 45 at WISC IV scale), with severe deficit language disturbance in production and understanding, and normal array-CGH and *FMR1* gene screening. Thyroid function and cardiac screening (cardiac US, ECG and Holter ECG) in the brother and the two sisters are normal (Figure 2).

### Genetic analysis

For WES, DNA libraries were enriched using Twist Human Core Exome Kit (Twist Bioscience), and sequenced on a NovasSeq6000 platform (Illumina, San Diego, CA). Bioinformatic analysis was performed as previously reported (14). Annotated variants were filtered and prioritized based on their quality, frequency in the population database gnomAD and predicted impact on the protein, with particular focus on genes implicated in CH and other thyroid defects. Identified variants were confirmed by Sanger sequencing. Accession numbers are NM_004387 for *NKX2-5* and NM_000369 for *TSHR*.

WES analysis detected potentially relevant heterozygous variants in two genes (Figure 2):

### NKX2-5 (*nucleotide kinase 2, homeobox 5*)

A recurrent missense variant Chr5(GRCh37):g.172662014G>A; c.73C>T, p.Arg25Cys, rs28936670 in *NKX2-5* exon 1 was found in the proband, his brother affected with ID and their mother. According to American College of Medical Genetics and Genomics (ACMG) criteria, the variant is classified as a variant of uncertain significance (VUS), and has a minor allelic frequency of 0,343% in the gnomAD database. This variant has already been reported in several patients with CHD as well as healthy individuals of various ethnicities (15,16).

### TSHR (*TSH receptor*)

The proband, his affected brother and two healthy sisters carried a heterozygous missense variant in *TSHR* Chr14(GRCh37):g.811534624_25AG>CT; c.269_270delinsCT; p.Gln90Pro inherited from their father. This variant is classified as likely pathogenic according to ACMG guidelines. No additional variants were detected in this gene.

## DISCUSSION

Here we report the co-occurrence of thyroid hemiagenesis, ventricular arrhythmias and intellectual disability in a young patient. In subjects with CH, the incidence of extra-thyroidal malformations is reported to be fourfold higher than in control population, the commonest associated defects being CHD (11,17). Whole exome sequencing and subsequent filtering detected potentially relevant heterozygous variants in two genes: *NKX2-5* and *TSHR.* Yet, genetic results, despite the consanguinity of the parents, were not conclusive and did not allow to reach a definite diagnosis, as explained below.

Heterozygous variants in the *NKX2-5* gene have been repeatedly reported in patients with CH and thyroid athyreosis, ectopy and hypoplasia, as well as with CHD (7–10). Mutations in this gene are estimated to account for approximately 4% of human cardiovascular malformations, most commonly secundum atrial septal defect (ASD), tetralogy of Fallot, and atrioventricular conduction disease (AVCD) (15,18–20).

The identified p.Arg25Cys variant is a common variant often identified in patients with conotruncal heart anomalies (15,16), but to the best of our knowledge, it has never been described in young patients with ventricular arrhythmias (Table S1) (7,8,15,16,19–25). Moreover, this variant was also reported in patients with thyroid ectopy, athyreosis and thyroid hypoplasia, but without cardiac malformations (7,8). Yet, the pathogenetic role of this variant is currently questioned, due to the high allelic frequency in specific populations (African-Americans) (15,16), as well as because of lack of segregation in families. Indeed, the incomplete penetrance and variable expressivity of this variant both at thyroidal and cardiac levels strongly suggest that other genes are expected to contribute to the phenotype.

**Table S1:** Previously reported patients and controls with the NKX2-5 p.Arg25Cys variant – Allelic frequencies are reported

References	Cardiac and/or thyroid anomaly		No. of patients carryng R25C	Family history	Other family members with R25C	No. Of control patients carryng R25C
Benson DW *et al.* (1999) (21)	TOF and VSD/no thyroid data		1/7	negative	not reported	0/50
Goldmuntz E *et al.* (2001) (15)	TOF/no thyroid data		3/114 (1,3%)	1 father: ASD	1 father	2/43 Africans Americans (2,3%)
McElhinney DB *et al.* (2003) (19)	7/608 (0,58%)no thyroid data	TOF Truncus arteriosus Interrupted aortic arch HLHS	1 1 1 1	father: VSD negative negative negative	not reported negative negative negative	0/50 randomcontrol Caucasian
Akcaboy MI *et al.* (2008) (16)	TOF/no thyroid data		1/72 (0,69%)	negative	father	2/185 Turkish (0,54%)
Stallmeyer B *et al.* (2010) (20)	HLHS/no thyroid data		1/121 (0,41%)	negative	negative	0/380 Caucasian
Perera JL *et al.* (2010) (4)	TOF/no thyroid data		1/159 (0,31%)	sister: VSD	brother	0/162 Brazilian
Rauch R *et al.* (2010) (22)	TOF/no thyroid data		2/230 (0,43%)	negative	negative	not tested
Beffagna G *et al.* (2013) (23)	2/100 (1%)	CoA, AVSD	1/100	negative	mother	0/250 Italian
		No thyroid defect				
		ASD, VSD	1/100	negative	not tested	
		Trisomy 21				
		No thyroid defect				
Dentice M *et al.* (2006) (7)	2/241 (0,41%)	Thyroid ectopy TSH 300 mU/L, FT4 5.4 pg/mL No cardiac malformation	1/241	negative	mother	1/561 (0,09%)
			Athyreosis	1/241	negative	father and brother
		TSH 419 mU/L, FT4 1.6 pg/mL				
		No cardiac malformation				
		Bilateral cortex atrophy				
		Attention deficit hyperactivity disorder				
Khatami M *et al.* (2017) (8)	2/65 (1,5%)	Thyroid hypoplasia TSH 17 mU/L, FT4 1.3 pg/mL No cardiac malformation	1/65	negative	negative	0/62 Iranian
	Thyroid hypoplasia TSH 21 mU/L, FT4 1.1 pg/mL No cardiac malformation		1/65	negative	negative	
Pulignani S *et al.* (2018) (24)	TOF no thyroid data		1/17	negative	negative	not tested
Alcantara-Ortigoza MA *et al.* (2021) (25)	1	Trisomy 21 and normal heart	3/148	negative	mother	1/113 Mexican
	1	Trisomy 21 and perimembranous VSD and PDA			father	
	1	Trisomy 21 and complete AVSD			mother negative, father not tested	
In this study	1	Ventricular arrhythmias, no cardiac malformation Thyroid hemiagenesis. TSH 740 mU/L, FT4 0.9 pmol/L Intellectual disability	1	brother: intellectual disability	mother	not tested

TOF: tetralogy of Fallot; VSD: ventricular septal defect; ASD: atrial septal defect; AVSD: atrioventricular septal defect; HLHS: hypoplastic left heart syndrome; CoA: aortic coartation. TSH normal range (0.35-4.5 mU/L), FT4 normal range (7.5-17.0 pg/mL, 12-22 pmol/L).

Functional studies on this variant also gave contrasting results. Arginine at codon 25 is conserved in mouse, rat and human, suggesting the potential deleterious consequences of a variation at this location. The residue lies just outside the first helix region of the functional domain; altering the charge from basic to neutral, the Cys25 variant may lead to a reduced hydrophobicity of the helix and the nearby region (16). Other functional studies showed for this variant a normal transcriptional activity and normal DNA binding to a monomeric site, but 3-fold reduction in DNA binding to dimeric sites, suggesting a slight impairment of dimerization (2), and normal DNA binding properties but impaired transactivation properties on target genes (7,26).

In our family, the variant was found in the proband as well as in the brother, presenting only ID but no cardiac or thyroid defects, and in the mother, whose autoimmune thyroiditis is likely to be completely unrelated to this genetic finding.

Besides this, the proband also carried a single heterozygous variant (p.Gln90Pro) in the *TSHR* gene, present also in the other siblings and the healthy father. This variant is classified as likely pathogenic, and functional *in vitro* studies mutation showed 55% lower activity compared with the wild type protein (27).

Recessive inactivating variants of the *TSHR* gene might manifest with a wide spectrum of thyroidal consequences: athyreosis at one end of the clinical range, mild hypothyroidism with normal thyroid gland size at the other far end (13). However, carriers of single heterozygous variants are healthy or present signs of subclinical hypothyroidism, i.e. hyperthyrotropinemia in euthyroidism (28). Indeed, the identified variant had already been reported in two large pedigrees of Arabic descend, in which some members also carried a mutation in the *TPO* gene encoding thyroid peroxidase. In these families, those who were solely heterozygous carriers of the *TSHR* variant were either euthyroid, or showed a mild subclinical hypothyroidism or a very mild hypothyroidism, without any sign of thyroidal dysgenesis (27).

While it is clear that none of the two variants is able to fully explain the thyroidal and cardiac manifestations of the proband, their potential contribution to the phenotype cannot be fully excluded and remains to be elucidated. Moreover, no relevant variants were identified that could potentially explain the intellectual disability in the patient and his similarly affected brother, as all variants surviving filtering in ID-related genes were all classified as variants of unknown significance and were inherited from one unaffected parent.

In conclusion, despite we acknowledge the great progress brought by large scale next generation sequencing to the diagnosis of complex genetic phenotypes, the patient reported here represents a paradigmatic example of how the interpretation of genetic variants can be an extremely difficult process, challenging genetic diagnosis and subsequent familial counselling.
